# Dysgeusia-Driven Nutritional Decline in Cirrhosis: A Case of Autoimmune Hepatitis and Primary Biliary Cholangitis

**DOI:** 10.7759/cureus.98842

**Published:** 2025-12-09

**Authors:** Newton Rahming, Stephan Corcho, Oluwasegun Olowe, Leila Falls, Murali K Manikkavelu, Frederick Tiesenga

**Affiliations:** 1 Surgery, Caribbean Medical University, Willemstad, CUW; 2 Surgery, St George's University, West Indies, GRD; 3 Surgery, Windsor University School of Medicine, West Indies, KNA; 4 General Surgery, American University of Antigua, St. John, ATG; 5 General Surgery, West Suburban Medical Center, Chicago, USA

**Keywords:** autoimmune hepatitis, child-pugh score, cirrhosis, dysgeusia, hepatic decompensation, malnutrition, mycophenolate mofetil, primary biliary cholangitis, taste disorders, zinc deficiency

## Abstract

Dysgeusia, or altered taste perception, is characterized by metallic, sweet, sour, or bitter sensations that can significantly impair oral intake and contribute to malnutrition, particularly in patients with chronic illness. This report describes a 75-year-old woman with decompensated cirrhosis secondary to autoimmune hepatitis and primary biliary cholangitis (PBC), who presented with a persistent metallic taste and an unintentional 25-pound weight loss over three months. Dysgeusia began shortly after the initiation of mycophenolate mofetil (MMF) and led to reduced caloric intake, early satiety, and progressive sarcopenia. Laboratory evaluation demonstrated pancytopenia, hypoalbuminemia, and hyperbilirubinemia, consistent with decompensated cirrhosis. Zinc levels were normal (82 mcg/dL), and the patient remained compliant with supplementation, making isolated zinc deficiency an unlikely cause of her symptoms. Longitudinal assessment demonstrated parallel declines in weight, nutritional intake, and hepatic synthetic function. Imaging and alpha-fetoprotein (AFP) testing excluded hepatocellular carcinoma, and no acute complications of cirrhosis were identified. The overall clinical picture suggested that dysgeusia-associated malnutrition, likely exacerbated by medication exposure in the context of portal hypertension, contributed substantially to hepatic decompensation. This case underscores the overlooked impact of taste disturbances in cirrhotic patients and highlights the importance of early identification, medication review, and nutritional intervention to mitigate malnutrition-related complications.

## Introduction

Dysgeusia is a distortion in taste perception that encompasses a wide range of sensory disturbances, including hypogeusia (reduced taste sensitivity), hypergeusia (heightened sensitivity), phantogeusia (phantom tastes), and aliageusia (distorted taste perception) [1]. Etiologies are multifactorial and include oral inflammation, micronutrient deficiencies, chemotherapy effects, medications, and chronic systemic diseases [1]. Despite its clinical relevance, dysgeusia is often underrecognized due to its subjective nature, lack of standardized diagnostic tools, and its perception as a non-urgent symptom [1].

In patients with chronic liver disease, dysgeusia can have substantial consequences. Malnutrition and sarcopenia occur in 65-95% of individuals with decompensated cirrhosis and are strongly associated with frailty, increased morbidity, and mortality [2,3]. Nutritional decline is further compounded by impaired appetite and reduced caloric intake, both of which contribute to disease progression. Hepatic dysfunction also affects micronutrient absorption, including zinc, a well-documented contributor to taste disturbances, yet not all cases of dysgeusia resolve despite zinc repletion [4-6].

Medication-induced dysgeusia is another important consideration in cirrhotic patients. Multiple commonly prescribed medications have been associated with taste disturbances, including diuretics, angiotensin-converting enzyme (ACE) inhibitors, proton pump inhibitors, and immunosuppressive agents [7,8]. These effects may occur through zinc chelation, altered salivary composition, or mucosal irritation [8,9]. Portal hypertension and its systemic manifestations may further exacerbate nutritional decline in advanced liver disease [10]. Clinical practice guidelines from the European Association for the Study of the Liver (EASL) emphasize that decompensated cirrhosis is frequently complicated by malnutrition, making the evaluation of taste disturbances increasingly relevant in routine care [11].

Although dysgeusia is widely recognized in the context of micronutrient deficiencies and certain medications, its role as a precipitating factor for malnutrition-related hepatic decline in autoimmune-related cirrhosis remains underreported. This case is documented to highlight how persistent dysgeusia occurring despite normal zinc levels and dose-adjusted immunosuppression can lead to significant caloric restriction, early sarcopenia, and worsening hepatic synthetic function. By detailing this presentation, we aim to increase clinical awareness of taste disturbances as an overlooked but modifiable contributor to nutritional deterioration in cirrhotic patients.

## Case presentation

A 75-year-old female with a known history of cirrhosis secondary to autoimmune hepatitis (AIH) and primary biliary cholangitis (PBC)-related cirrhosis presented on September 2025 for a persistent metallic taste and frequent falls ongoing for about four months. The patient reported a metallic taste began approximately two weeks after starting mycophenolate mofetil (MMF) 1000mg every 12 hours. This taste alteration progressively worsened and impaired her food and water intake. This nutritional restriction negatively affected her overall health, resulting in a progressive caloric deficit, early satiety, and significant unintentional weight loss. No changes reported to her prescribed medication regimen. The patient’s recorded weight was 91.6kg (202 lbs), which is an 11.1 kg (25 lbs) loss compared to the patient's reported weight four months prior. This nutritional deficit impacted her BMI from 36.6 to 32.6, representing an 11% reduction in total body weight within a span of four months. This degree of weight loss portrays high clinical significance for concern of a worsening malnutrition trajectory especially with respect to the need for this patient’s nutritional adequacy due to underlying medical conditions.

At admission she was found to be hypokalemic with levels of 2.6 mmol/L and was hypertensive. No new reported cardiac abnormalities were recorded at this time. Patient workup included discontinuation of hydrochlorothiazide, zinc supplementation, initiation of amlodipine and aggressive repletion of potassium fluids with normalization on the day of discharge. Discharged medications included ursodiol 600 mg three times daily, titrated MMF 1000 mg twice daily to 500 mg twice daily due to complaints of metallic taste, and amlodipine. 

Upon laboratory tests, the patient was found to be hypokalemic (2.9 mmol/L), hypoproteinemic (5.6 g/dL), hypoalbuminemic (2.4g/dL) and had elevated liver function tests (LFTs) (aspartate aminotransferase (AST): 108 U/L, alkaline phosphatase (ALP): 302 U/L). Physical exam findings indicated ascites and bilateral leg edema. Nuclear medicine (NM) hepatobiliary report showed gallbladder ejection fraction of 7% indicating biliary dyskinesia. The patient was managed with electrolyte repletion, spironolactone 12.5 mg daily, and furosemide 20 mg daily. With consultation, this patient was rendered moderate risk with reference to the Child Pugh classification. 

The patient reports no prior history of spontaneous bacterial peritonitis (SBP), variceal bleeding, gastrointestinal hemorrhage, hepatic encephalopathy, or hepatocellular carcinoma. A previous esophagogastroduodenoscopy (EGD) in 2015 demonstrated portal hypertensive gastropathy without varices. 

Patient medication list at presentation included aspirin, budesonide, carvedilol, ergocalciferol, lisinopril, MMF, nifedipine, pantoprazole, rosuvastatin, spironolactone, furosemide, vitamin A, zinc sulfate, ursodiol, oral potassium chloride, and periodic intravenous potassium chloride supplementation. Oral and ENT examinations were unremarkable, with no evidence of mucosal lesions, candidiasis, ulcerations, or xerostomia. A formal nutrition assessment revealed a daily caloric intake of approximately 800 kcal/day with reduced protein consumption. Grip strength testing demonstrated decreased muscle strength, consistent with early sarcopenia. These findings supported significant malnutrition secondary to dysgeusia, contributing to reduced metabolic reserve in the setting of chronic liver disease.

During her hospitalization and subsequent outpatient follow-up, the patient received nutritional counseling, electrolyte repletion, and medication review. MMF was reduced to 500 mg twice daily due to suspected contribution to her taste disturbance. Despite the dose adjustment, dysgeusia persisted, though she reported a mild improvement in appetite with structured meal planning and high-calorie oral supplements. Over the following eight weeks, she regained approximately four pounds and demonstrated improved grip strength, though albumin levels remained low. No episodes of variceal bleeding, encephalopathy, or spontaneous bacterial peritonitis occurred during follow-up. She continues routine hepatology care with ongoing nutritional monitoring.

Admission laboratory studies are summarized in Table 1. Blood tests revealed anemia, thrombocytopenia, leukopenia, hypoalbuminemia, hyperbilirubinemia, and elevated international normalized ratio (INR) indicative of decompensated cirrhosis. Zinc level was within normal range.

**Table 1:** Key laboratory findings on presentation. Laboratory results demonstrate normal zinic, pancytopenia, hypoalbuminemia, and hyperbilirubinemia, consistent with decompensated cirrhosis. INR: international normalized ratio; PT: prothrombin time.

Blood Test	Result	Reference Range
Hemoglobin	11.6 g/dL	12-15 g/dL
Platelets	62 * 10^9/L	150-400 x 10^9/L
White Blood Cells	1.7 * 10^9/L	4.5-11.0 x 10^9/L
Albumin	2.4 g/dL	3.5-5.5 g/dL
INR/PT	1.3/14.5	0.8-1.1
Bilirubin	1.7 mg/dL	0.1-1.2 mg/dL
Zinc	82 mcg/dL	70-120 mcg/dL

To enhance clarity regarding the patient’s clinical trajectory, a longitudinal summary of laboratory trends, weight and BMI changes, nutritional status, and medication adjustments was compiled. This provides a concise visualization of her progressive metabolic decline and its relationship to dysgeusia-induced malnutrition.

As shown in Table 2, the patient’s progressive weight loss, decreasing albumin levels, and reduced nutritional intake paralleled the worsening of her dysgeusia, supporting malnutrition as a significant contributor to her decompensation.

**Table 2:** Longitudinal trend of hepatic function and nutritional status during clinical decline. This table summarizes longitudinal changes in weight and BMI, laboratory markers of hepatic function, nutritional intake estimates, and medication adjustments. Alb: albumin, Bili: bilirubin, INR: international normalized ratio, Plt: platelets, MMF: mycophenolate mofetil, BID: twice a day

Timepoint	Weight (lb) / BMI	Key Labs (Alb, Bili, INR, Plt)	Nutritional Indicators	Medication Changes
Baseline (3 mo prior)	227 lb (BMI 36.6)	Alb 3.4 g/dL, Bili 1.7 mg/dL, INR 1.3, Plt low	Normal appetite; no caloric deficit; no sarcopenia	MMF initiated 1000 mg BID
Month 1	219 lb	Alb 3.1, Bili ↑, INR stable, Plt ↓	↓ appetite; ~1,200 kcal/day; early sarcopenia	MMF reduced to 500 mg BID
Month 2	210 lb	Alb 2.8, Bili ↑, WBC/Plt ↓, INR 1.3	~1,000 kcal/day; worsening grip strength	Spironolactone + furosemide
Presentation	202 lb (BMI 32.6)	Alb 2.4, Bili 1.7, INR 1.3, Plt 62	~800 kcal/day; early sarcopenia; reduced protein intake	Spironolactone + furosemide

A multidisciplinary evaluation revealed no acute intracranial pathology on MRI, no cervical spine injury and a normal echocardiogram with preserved left ventricular function. Duplex ultrasound ruled out deep venous thrombosis. Ultrasound of the right upper quadrant performed for hepatocellular carcinoma (HCC) surveillance demonstrated a cirrhotic liver morphology without focal hepatic lesions, with no sonographic evidence of hepatocellular carcinoma. 

Finally, viral serology was negative for hepatitis A, B, and C, and the tumor marker for alpha-fetoprotein (AFP) was negative, making viral hepatitis and hepatocellular carcinoma less likely as underlying etiologies for her decompensation and taste disturbance.

## Discussion

This case report highlights that dysgeusia-induced malnutrition can be a primary driver of hepatic decompensation in cirrhotic patients, a relationship often overlooked in clinical practice and not fully explained by common etiologies such as zinc deficiency. Dallio et al. identified a strong linear relationship between dysgeusia and liver disease progression, recommending routine monitoring of altered taste in patients with hepatic conditions [1].

Malnutrition is highly prevalent in cirrhosis and is strongly associated with frailty, sarcopenia, and increased morbidity and mortality [2]. The American Association for the Study of Liver Diseases (AASLD) practice guidance further emphasizes that malnutrition and sarcopenia are major prognostic factors and are often underrecognized in routine care [3]. Micronutrient deficiencies, including zinc and vitamins A and B complex, are common in cirrhosis due to altered metabolism, poor intake, and impaired absorption [4]. Zinc deficiency specifically contributes to dysgeusia by disrupting carbonic anhydrase VI activity in saliva, impairing taste bud function [5]. Although randomized and observational studies have shown that zinc supplementation can improve taste perception and nutritional status in cirrhotic patients [5,6], our patient had normal zinc levels and remained compliant with supplementation, making zinc deficiency an unlikely etiology.

Medication effects represent another key consideration in dysgeusia, particularly in patients with polypharmacy or impaired hepatic metabolism. Drug-induced taste disturbances have been documented across a wide range of agents, including diuretics, ACE inhibitors, and proton pump inhibitors [8]. Mechanisms include zinc chelation, altered salivary composition, and mucosal irritation [9]. These effects, combined with impaired hepatic metabolism, can exacerbate sensory alterations in cirrhotic patients. Thiazide diuretics, which the patient previously used, have also been shown to increase zinc excretion and contribute to altered taste, though the delayed onset and discontinuation make this less likely.

MMF, a potent immunosuppressant commonly used in autoimmune liver disease, has been associated with dysgeusia in isolated post-marketing reports, although robust evidence remains limited [8]. In this case, the persistence of dysgeusia despite a 50% dose reduction suggests either a dose-independent adverse effect or a multifactorial etiology. Further study is warranted to clarify the mechanisms underlying MMF-associated taste disturbances.

Cirrhosis-related factors also contributed to this patient’s hepatic deterioration. She exhibited portal hypertension with hypersplenism, pancytopenia, and mild to moderate ascites, classic features of advancing decompensation [10]. EASL clinical practice guidelines similarly emphasize that decompensated cirrhosis is frequently complicated by nutritional decline and loss of hepatic reserve [11]. Her sustained caloric deficit reduced protein intake, and sarcopenia likely interacted with underlying portal hypertension, further impairing hepatic synthetic function and accelerating decompensation.

Given the patient’s clinical decline, evaluation for HCC was appropriately performed. Both imaging and AFP levels were normal, consistent with AASLD HCC guidance, which recommends routine surveillance in cirrhotic patients [12]. Thus, malignant transformation was unlikely to be the cause of her decompensation.

Assessment of medication-related adverse effects may be supported by tools such as the Naranjo Adverse Drug Reaction Probability Scale, although this was not administered in this case [13]. Nevertheless, the temporal association with MMF initiation raises the possibility of a drug-related contribution to dysgeusia.

Persistent dysgeusia and the resulting decline in oral intake likely contributed to the patient’s progressive weight loss and worsening hepatic status. Her Child-Pugh Class B score reflects moderate hepatic dysfunction and an elevated risk of decompensation-related complications [14]. The specific scoring values used to determine this classification are summarized in Table 3. Taste alterations have also been strongly associated with unintentional weight loss, malnutrition, and reduced quality of life in cirrhotic patients [15], aligning with this patient’s 25-pound weight loss over a three-month period.

**Table 3:** Modified Child-Pugh Score Calculation for Decompensated Cirrhosis. The patient’s total score of 8 corresponds to Child-Pugh Class B, indicating moderate hepatic dysfunction. INR: international normalized ratio

Parameter	Patient Value	Score
Encephalopathy	None	1
Ascites	Mild-Moderate	2
Bilirubin	1.7 mg/dL	1
Albumin	2.4g/dL	3
INR	1.3	1
Total Score	-	8

Overall, this case underscores the importance of recognizing dysgeusia as a contributor to malnutrition and hepatic decompensation. Early identification, medication review, nutritional support, and multidisciplinary management are essential in mitigating its impact in patients with chronic liver disease.

## Conclusions

This case illustrates that dysgeusia can significantly worsen nutritional status in patients with autoimmune-related cirrhosis, even in the absence of micronutrient deficiencies. Persistent taste disturbance following initiation of mycophenolate mofetil contributed to reduced caloric intake, early sarcopenia, and worsening hepatic reserve in this patient. Clinicians should consider dysgeusia as a potential early marker and modifiable contributor to malnutrition, prompting closer nutritional assessment and medication review in cirrhotic patients. Our findings highlight that dysgeusia-induced malnutrition can act as a precipitating factor for hepatic decompensation, warranting greater clinical attention in the care of patients with chronic liver disease. Future studies should explore structured screening tools and targeted nutritional interventions to improve outcomes in this vulnerable population.
